# T-Cell Manipulation Strategies to Prevent Graft-Versus-Host Disease in Haploidentical Stem Cell Transplantation

**DOI:** 10.3390/biomedicines5020033

**Published:** 2017-06-21

**Authors:** Jayakumar Vadakekolathu, Sergio Rutella

**Affiliations:** John van Geest Cancer Research Centre, Nottingham Trent University, Clifton Lane NG11 8NS, UK; jayakumar.vadakekolathu@ntu.ac.uk

**Keywords:** graft-versus-host disease, haploidentical haematopoietic stem cell transplantation, regulatory T cells, immunomagnetic T-cell depletion, photodynamic purging, dendritic cells

## Abstract

Allogeneic haematopoietic stem cell transplantation (HSCT) from an human leukocyte antigen (HLA)-identical donor can be curative for eligible patients with non-malignant and malignant haematological disorders. HSCT from alternative donor sources, such as HLA-mismatched haploidentical donors, is increasingly considered as a viable therapeutic option for patients lacking HLA-matched donors. Initial attempts at haploidentical HSCT were associated with vigorous bidirectional alloreactivity, leading to unacceptably high rates of graft rejection and graft-versus-host disease (GVHD). More recently, new approaches for mitigating harmful T-cell alloreactivity that mediates GVHD, while preserving the function of tumour-reactive natural killer (NK) cells and γδ T cells, have led to markedly improved clinical outcomes, and are successfully being implemented in the clinic. This article will provide an update on in vitro strategies and in vivo approaches aimed at preventing GVHD by selectively manipulating key components of the adaptive immune response, such as T-cell receptor (TCR)-αβ T cells and CD45RA-expressing naive T cells.

## 1. Introduction

Although human leukocyte antigen (HLA)-matched related donors (MRD) remain the preferred source of stem cells for allogeneic haematopoietic stem cell transplantation (HSCT), only 25% of the patients will locate a fully HLA-matched sibling [1]. Also, the identification of a complete HLA-matched unrelated donor (MUD) for HSCT remains a challenge, despite the availability of large international donor registries. Alternative donor sources are increasingly being considered and include HLA partially-matched or haploidentical family donors, i.e., related donors who share with the patient a single identical copy of chromosome 6, containing the HLA loci [2].

The failure of engraftment and graft-versus-host disease (GVHD) are major hurdles to the success of allogeneic HSCT. Graft rejection can be attributed to major disparities in HLA loci between the donor and recipient, resulting in an undesired immune response mounted by the host’s immune system. GVHD is the consequence of donor immune cells attacking the host, leading to extensive tissue damage and, in many instances, life-threatening complications and patient death [3]. Clinical GVHD is conventionally classified as acute GVHD (if occurring within the first 100 days after HSCT) and chronic GVHD (which occurs after day 100 post-HSCT). Immune suppressive treatments are a cornerstone in GVHD prophylaxis, as reviewed elsewhere [3]. Novel approaches to preserve the beneficial anti-leukaemia effects of donor T cells without inducing detrimental GVH responses are needed to maximise the therapeutic potential of allogeneic HSCT. Despite the complexities relating to the mechanistic understanding of GVHD and its treatment [4], allogeneic HSCT remains a highly successful and curative modality for haematological malignancies and inherited or acquired non-malignant blood disorders, as well as for an expanding number of autoimmune diseases [5].

**Figure 1:**
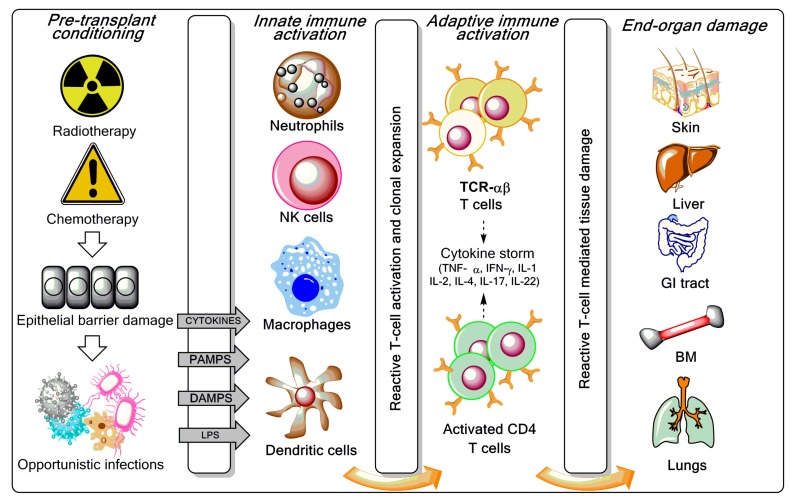
Overview of graft-versus-host disease (GVHD) pathogenesis. As reviewed elsewhere [3,7,8], the pre-transplant conditioning regimen (chemotherapy with or without radiotherapy) contributes to GVHD induction via tissue destruction, bacterial translocation across gut mucosal cells, and release of pro-inflammatory cytokines such as tumour necrosis factor (TNF)-α, interleukin (IL)-1, and IL-7. Lipopolysaccharide (LPS) and other components of the bacterial cell wall leaking through the damaged intestinal mucosa stimulate mononuclear cells, amplify the production of inflammatory cytokines, and contribute to apoptosis. Innate immune cells partake in tissue damage and cytokine production, a phenomenon referred to as “cytokine storm”. Both host and donor antigen-presenting cells (APCs) initiate GVH responses through the release of IL-12 and IL-23. Activated T cells, natural killer (NK) cells, and macrophages mediate end-organ damage through cytokine production and direct cytotoxic effects on target tissues (release of cytolytic granules and expression of the CD95 ligand). Acute GVHD develops in parenchymal targets containing highly proliferating cells (bone marrow, skin, liver, gut, and lungs). CD4^+^ T cells are also activated by macrophages and dendritic cells (DCs) to produce pro-inflammatory mediators. CTL = cytotoxic T lymphocyte; IFN = interferon; PAMP = pathogen-associated molecular pattern; DAMP = damage-associated molecular pattern; TCR = T-cell receptor; BM = bone marrow; GI = gastrointestinal.

A major obstacle to three-loci-mismatched haploidentical HSCT is the HLA barrier, mainly in the GVH direction, and bidirectional alloreactivity (Figure 1). Therefore, various approaches to prevent GVHD are being investigated, including in vitro T-cell depletion of bone marrow or peripheral blood stem cells (PBSCs) or, more recently, in vivo T-cell depletion approaches using either granulocyte colony-stimulating factor (G-CSF)-mobilized bone marrow in combination with PBSCs and anti-thymocyte globulin or the administration of high-dose cyclophosphamide after transplantation of haploidentical bone marrow-derived progenitor cells [6].

GVHD is a multistage process incited by pre-transplantation conditioning regimens (radiation and/or chemotherapy) [9]. Increased tissue damage and generation of pathogen-associated molecular patterns (PAMPs) and damage-associated molecular patterns (DAMPs) trigger danger-recognising receptors, such as the Toll-like receptors (TLRs), and fully activate antigen presenting cells (APCs) (Figure 1), leading to the secretion of pro-inflammatory cytokines, as well as the activation and expansion of donor T cells. Trafficking of donor T cells towards target sites of GVHD is the second stage in GVHD pathogenesis, where chemokines and other inflammatory mediators released as a result of pre-transplantation conditioning regimens play a critical role. Tissue damage is mainly effected by cytotoxic T cells and FAS (CD95)-FAS ligand (CD95L) or perforin/granzyme-mediated pathways. Other cell types, such as natural killer (NK) cells, partake in GVHD initiation, as reviewed elsewhere [9].

Despite being a major clinical challenge, mild-to-moderate GVHD might have a therapeutic effect, commonly referred to as graft-versus-leukaemia (GVL), which mainly results from the recognition and killing of tumour cells by donor-derived alloreactive T cells. Different approaches have been developed in addition to pharmacological interventions to dampen GVH while preserving GVL responses, including the administration of genetically-modified T cells, dendritic cells (DCs), and NK cells, and the co-administration of mesenchymal stromal cells, as reviewed elsewhere [3,9,10].

This article focuses on the latest developments in graft engineering techniques to target T cells with the aim of reducing GVHD while preserving anti-tumour and anti-pathogen immune responses after haploidentical HSCT.

## 2. Depletion of T-Cell Subpopulations

**Figure 2:**
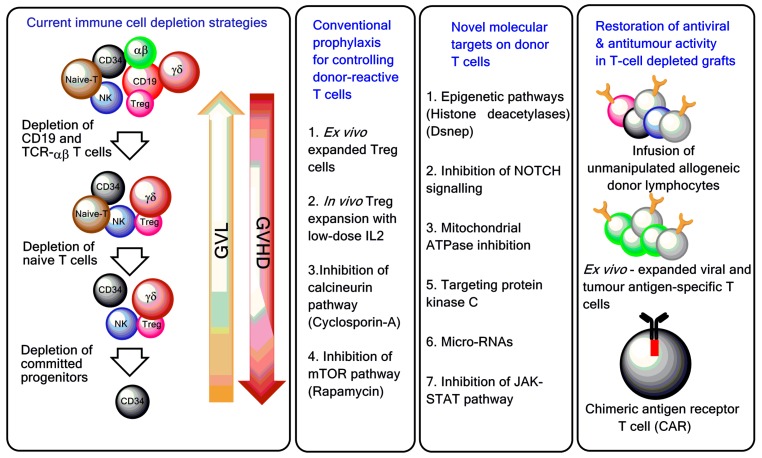
T-cell manipulation strategies to control GVHD and improve post-haematopoietic stem cell transplantation (HSCT) immune reconstitution. Ex vivo T-cell depletion techniques have evolved and currently include CD3/CD19 depletion, TCR-αβ/CD19 depletion, and infusion of CD45RA-depleted grafts [14,15,16]. To boost immune reconstitution after T-cell depleted haploidentical HSCT, genetically modified T cells and pathogen-specific T cells are increasingly used in the clinic [17,18]. Treg = regulatory T cell; GVL = graft-versus-leukaemia; mTOR = mechanistic target of rapamycin; NOTCH = neurogenic locus notch homolog protein; JAK-STAT = Janus kinase/signal transducer and activator of transcription.

A major breakthrough in GVHD prevention was the depletion of donor T cells by physical or immunological techniques [11], including soybean lectin agglutination, rosette depletion, and monoclonal antibody-mediated methods. The development of preclinical models of transplantation of mega-doses of highly purified CD34^+^ progenitor cells described by Reisner et al. [12], and their subsequent application to patients by Aversa et al. [13], were major steps towards a broader clinical use of haploidentical HSCT. The introduction of the one-step, semi-automated MACS^®^ device (Clini-MACS, Miltenyi Biotec, Bergisch Gladbach, Germany) brought further improvement, including the achievement of a median CD34-cell purity of 97% and an extensive depletion of T and B cells (Figure 2). Pioneering clinical trials with the Miltenyi semi-automated device were performed in children and showed a high engraftment rate with a low incidence of acute and chronic GVHD (reviewed in [6]).

### 2.1. Depletion of T-Cell Receptor (TCR)-αβ T Cells and CD19^+^ B Cells

In order to avoid extensive GVHD and to prevent post-transplantation Epstein-Barr virus (EBV)-induced lymphoproliferative disease (EBV-PTLD), CD3/CD19-depleted HLA-haploidentical HSCT has been evaluated within a prospective phase II trial in children with acute leukaemia and advanced myelodysplastic syndrome, who received a myeloablative conditioning regimen consisting of fludarabine or clofarabine (in patients with active disease only), thiotepa, melphalan, and serotherapy [15]. Using this graft manipulation procedure, the number of CD3^+^ T cells remained below the threshold value of 1 × 10^5^ cells/kg of the recipient’s body weight, even in small children weighing less than 10 kg. Transplantation-related mortality (TRM) was 8% at 1 year and 20% at 5 years post-transplant. Primary engraftment was documented in 87% of children, with 1000/µL leukocytes and 20,000/µL platelets being reached 10 and 11 days after HSCT, respectively. Acute grade II and grade III-IV GVHD occurred in 26% and 6.5% of patients, respectively, whereas chronic GVHD was observed in 21% of evaluable patients. The cumulative incidence of cytomegalovirus (CMV) re-activation was 21% for all patients and 52% and 0% for recipient (R)^+^donor (D)^+^/R^+^D^−^ and R^−^D^+^/R^−^D^−^ subgroups, respectively, whereas the cumulative incidence of human adenovirus (HAdV)-DNA in stool was 53%. Notably, in most patients, an endogenous T-cell response to viral antigens was detected, with HAdV-reactive and CMV-reactive T cells being detected in vitro at a median frequency of 0.12% and 0.6%, respectively, after stimulation with hexon antigen/virus lysate and pp65. Pulmonary aspergillosis was diagnosed in 6 patients (proven in 1, probable in 5); 3 out of these 6 patients had pre-existing infiltrates prior to HSCT conditioning. However, no lethal fungal or viral infection occurred in any of the patients. The median time to reach >0.1 × 10^9^/L CD3^+^CD4^+^ and CD3^+^CD8^+^ T cells was 61 and 107 days, respectively. By contrast, the recovery of CD56^+^ NK cells was prompt, with co-transfused NK cells being detectable in the first week after HSCT. Event-free survival (EFS) at 3 years for the whole group was 25% [15]. Remission status significantly influenced survival, with patients transplanted with active disease having a 3-year EFS of 15%. However, a subgroup analysis of patients who received a first haploidentical HSCT in the first, second, or third complete remission showed a favourable 3-year EFS of 46% [15]. The overall probability of relapse at 2 years was 38% in patients receiving the first HSCT in complete remission and 75% in those who were transplanted with the active disease.

A TCR-αβ depletion reagent has been available for the Clini-MACS system since 2009. To maintain additional anti-tumour and anti-infectious properties in the graft, a novel strategy aimed at depleting TCR-αβ^+^ T cells and B cells has been recently validated (Table 1) [14]. The authors performed 102 separations with a 4.7-log and 4.1-log depletion of TCR-αβ^+^ and CD19^+^ cells, respectively. Comparison with other techniques of T-cell depletion, including positive selection of CD34^+^ cells and CD3/CD19 depletion, revealed a comparable or better performance in terms of CD34 enrichment and CD3/CD19 depletion, with more constant results and lower coefficients of variation [14].

Comprehensive 10-colour flow cytometry panels have recently been developed for quality control purposes and to allow for the correct application of desired stem cell and T-cell dosages for clinical-scale TCR-αβ/CD19-depleted grafts [25].

**Figure 3:**
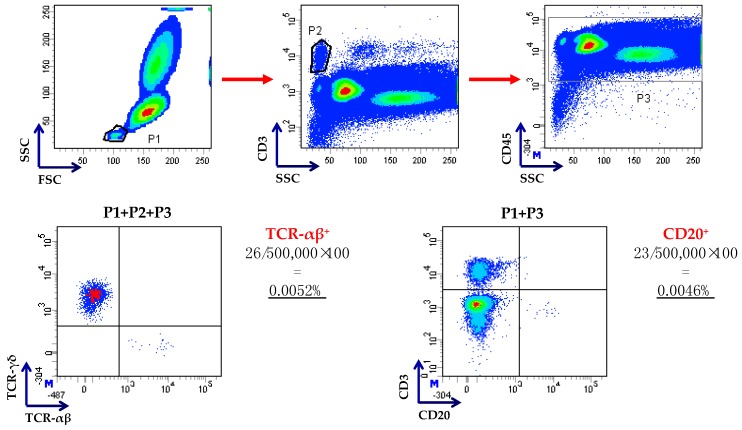
Flow cytometry-based enumeration of residual T and B cells after TCR-αβ/CD19 depletion. Cells were gated on low side scatter/low forward scatter events (P1), followed by gating on CD3^+^ T cells (P2) and on CD45^+^ leukocytes (P3), as already published [22]. Residual TCR-αβ^+^ and CD20^+^ B cells were enumerated as shown in this representative procedure. Anti-CD20 monoclonal antibodies were used because of strong internalization of CD19 after the incubation of cells with CD19 reagent or steric hindrance by the beads, as suggested in [14].

Using the same approach (Figure 3), 23 children were treated with HLA-haploidentical HSCT for non-malignant disorders, without receiving any post-transplantation pharmacological GVHD prophylaxis [16]. The median number of CD34^+^ cells, TCR-αβ^+^ T cells, and B cells infused was 16.8 × 10^6^/kg, 40.0 × 10^3^/kg, and 40.0 × 10^3^/kg, respectively. All patients but 4 engrafted. Three patients developed grade I–II acute GVHD of the skin, with no visceral acute or chronic GVHD being reported. The cumulative incidence of TRM was 9.3%. With a median follow-up of 18 months at the time of study publication, 21/23 children are alive and disease-free, with the 2-year probability of disease-free survival (DFS) being 91.1%. The recovery of γδ^+^ T cells was prompt, whereas αβ^+^ T cells progressively increased over time.

A retrospective study involving 34 adult leukaemia patients (AML and ALL) who received TCR-αβ-depleted haploidentical HSCT with CD34^+^ progenitors after a myeloablative conditioning regimen showed favourable GVHD-free survival rates [23]. Full engraftment and donor chimerism were established in 31 patients, and grade III–IV acute GVHD and chronic GVHD developed in four patients. One-year overall survival and disease-free survival were 54% and 42%, respectively. The only risk factor identified for overall survival was relapse, which was higher in ALL compared with AML patients. Neither EBV-PTLD nor CMV-related complications were observed in any patient with immune reconstitution.

B-cell reconstitution was not dissimilar to that observed after allogeneic HSCT [26], with >200 B cells/μL of blood being detected 4 months and 1 year after the infusion of TCR-αβ^+^/B-cell depleted grafts for primary immune deficiencies and acute leukaemia, respectively [16,19,20,21]. Further studies with larger cohorts of patients are needed to characterise long-term B-cell reconstitution in children and adults receiving extensively T-cell/B-cell depleted haploidentical grafts.

Overall, the studies published thus far point to the safety and efficacy of this graft manipulation approach for HLA-haploidentical HSCT, although refined treatment options to boost immunological reconstitution and to maintain long-term disease remission are needed.

### 2.2. Depletion of Naïve T Cells

Most T cells that cause GVHD reside within the naïve T-cell subset, unless the donor has developed a memory T-cell response through exposure to allogeneic cells after either pregnancy or blood product transfusion [27]. A single-arm first-in-human clinical trial enrolling 35 patients with high-risk leukaemia has shown that transplantation with >5.0 × 10^6^ CD34^+^ cells/kg and a defined dose of naive CD45RA T-cell-depleted peripheral blood stem cell (PBSC) grafts after a total body irradiation (TBI)-containing myeloablative conditioning regimen translates into prompt engraftment [28] (Table 2). An average number of 3600 naïve T cells/kg was infused. Although the incidence of grade II-IV acute GVHD was 66%, GVHD was always responsive to corticosteroids. The estimated probability of chronic GVHD was 9% at 2 years compared with 50% rates in a contemporary cohort of patients receiving T cell-replete grafts. Rapid T-cell recovery and transfer of protective virus-specific immunity could be documented, and overall survival was 78% at 2 years [28].

A novel 2-step, good manufacturing practice (GMP)-compliant procedure to deplete naïve T cells, while preserving CD34^+^ HSCs and pathogen-specific memory T cells, has been recently developed [29]. CD34^+^ HSCs were initially selected from G-CSF-mobilized apheresis products, followed by depletion of CD45RA^+^ cells using a murine anti-CD45RA monoclonal antibodies directly conjugated to iron dextran beads. A theoretical advantage of CD45RA depletion, as compared with complete T-cell depletion (TCD), is that pathogen-specific memory T cells are retained in the graft and could transfer protective immunity to opportunistic pathogens, such as CMV and EBV. In addition to a 4.5–5.0-log depletion of naïve T cells, CD45RA-depleted products contained a lower number of regulatory T cells (Treg), B cells, and NK cells, all of which express CD45RA. Importantly, the frequency of multifunctional virus-specific CD4^+^ and CD8^+^ T cells was equivalent or even higher in CD45RA-depleted products compared with un-manipulated grafts. The cost of the 2-stage cell selection procedure was considerable and estimated to be approximately $20,000, potentially limiting a broad application of this methodology. However, if effective at preventing GVHD while sparing pathogen-specific immunity, this approach might compare favourably with other TCD methods, including the removal of TCR-αβ^+^ T cells and CD19^+^ B cells.

### 2.3. Depletion of Alloreactive T Cells

Photodynamic allodepletion aims at eliminating host-reactive donor T cells from allogeneic HSCT to prevent GVHD, while conserving donor immunity [30]. This clinical-scale, semi-closed process is based on three consecutive phases, i.e., coloration, extrusion, and light exposure, and targets activation-based changes in P-glycoprotein, which result in an altered efflux of the photosensitizer TH9402. Lymphocytes expanded with anti-CD3 and IL-2 served as APCs and were co-cultured with responder cells from HLA-matched or mismatched donors. In mismatched stimulator-responder pairs, alloreactivity was reduced by a median 474-fold compared with the un-manipulated responder cells. By contrast, third-party responses were maintained. In matched pairs, alloreactive helper T-lymphocyte precursors were reduced to <1:100,000, while third-party responses remained around or >1:10,000. Specific anti-viral and anti-bacterial immunity was preserved in photo-depleted products.

In a clinical trial designed to evaluate the efficacy of photo-depletion to prevent severe acute GVHD, 24 patients with haematological malignancies conditioned with fludarabine, cyclophosphamide, and TBI received a CD34-selected allograft from a MRD along with 5 × 10^6^/kg photo-depleted donor T cells [31,32]. The photo-depleted product showed an inverted CD4^+^/CD8^+^ ratio, with the greatest depletion occurring in CD4^+^ naive and central memory T-cell subsets. By contrast, CD8^+^ naive and effector cells were relatively unaffected, reflecting the differential retention of TH9402 by T-cell subsets, which was greater in CD4^+^ and central memory cells and led to their preferential elimination during the photo-depletion procedure [32]. Engraftment was rapid, with 95% donor myeloid chimerism occurring by day 14, and 95% donor T-cell chimerism occurring by day 30 in most patients. Probabilities of acute GVHD were 38% for grades II–IV and 13% for grades III–IV, and 65% for chronic GVHD. The probability of haematological relapse was low (27%), considering the high-risk characteristics of the patient population. In multivariable analysis, low CD4^+^ central memory frequency in photo-depleted products was associated with chronic GVHD and worse overall survival (OS) [32]. An unexpected outcome of this study was the high rate of late viral, bacterial, and fungal infections that accompanied chronic or persisting acute GVHD, with 53% non-relapse mortality and median survival of only 568 days. In vitro proliferative responses to CMV were significantly reduced in the photo-depleted product, and reactivity to CMV remained much lower than in a control cohort of 35 patients given TCD HLA-matched transplants [32].

A cell-based therapeutic consisting of photo-depleted lymphocytes, ATIR^TM^ (Allodepleted T-cell ImmunotheRapeutic), has been developed by Kiadis Pharma. An open-label, multi-centre phase 2 study (CR-AIR-007; NCT01794299) enrolled 23 patients with acute leukaemia who received haploidentical CD34^+^ cells followed by a fixed dose of 2 × 10^6^/kg photo-depleted donor lymphocytes (ATIR101) at a median of 28 days after HSCT, without the use of post-transplant GVHD prophylaxis [33]. Ex vivo photo-depletion translated into the inactivation of host tissue-specific, proliferating T cells while maintaining anti-third party and anti-CD3/CD28 reactivity. No grade III/IV GVHD was observed after the infusion of allo-depleted T cells. Furthermore, 1-year OS was higher in patients receiving ATIR101 compared with a historic control group (61% in the HSCT + ATIR101 group vs. 20% in the control group). As of August 1st, 2016, two patients relapsed within the first year after HSCT. Severe infections occurred in 9/23 patients within the first 6 months after HSCT, which were viral infections in 7/23 subjects. GVHD-free, relapse-free survival (GRFS) for patients receiving HSCT + ATIR101 was estimated to be 57% at 1 year after HSCT, which compares favourably with the control group of patients receiving TCD-HSCT only (20%).

Overall, this TCD strategy is promising, although further pre-clinical development is warranted to avoid the undesired elimination of clinically useful T-cell subsets that contribute to post-transplantation immune recovery and control of infections.

In vivo depletion of alloreactive T cells with high-dose cyclophosphamide administered early post-transplantation (PTCy) allows the engraftment of haploidentical grafts from either bone marrow or peripheral blood without severe acute and chronic GVHD [36]. Outcomes for the first patients transplanted at Johns Hopkins were published in 2002 [37]. This strategy has been used in patients receiving tacrolimus and mycophenolate as GVHD prophylaxis and following reduced-intensity or non-myeloablative conditioning. Although this approach is technically less demanding and considerably more affordable compared with in vitro T-cell depletion, studies have shown ~50% relapse rates 1 year after transplantation [38], which could be the result of alloreactive T-cell depletion and reduced GVL responses. More recently, haploidentical bone marrow transplantation (BMT) with PTCy was shown to yield similar survivals to those observed in patients receiving HLA-matched BMT [39]. When patients in this retrospective analysis were risk-stratified using the Disease Risk Index (DRI), low-risk, intermediate-risk, and high/very high-risk patients had 3-year progression-free survival (PFS) estimates of 65%, 37%, and 22%, with corresponding 3-year OS estimates of 71%, 48%, and 35%, respectively [39]. Other investigators are attempting to further reduce relapse rates after haploidentical BMT with PTCy by using myeloablative busulfan and fludarabine conditioning [40]. However, non-relapse mortality at 100 days and 1 year were 9% and 16%, respectively, predominantly as a result of infections. Another encouraging report of 148 patients with a variety of haematological malignancies showed a 13% cumulative incidence of TRM after unmanipulated haploidentical BMT with PCTy [41,42]. The cumulative incidence of grades II–IV acute GVHD, grades III–IV acute GVHD, and chronic GVHD were 24%, 10%, and 12%, respectively.

## 3. Treatment of Donors to Prevent Graft-Versus-Host Disease (GVHD)

### 3.1. Granulocyte Colony-Stimulating Factor (G-CSF)

Experimental observations in the mid-1990s linked G-CSF to immune deviation in humans. G-CSF promotes the differentiation of type 1 Treg cells (Tr1) cells, endowed with the ability to release interleukin (IL)-10 and transforming growth factor (TGF)-β1, and to suppress T-cell proliferation in a cytokine-dependent manner [43]. Finally, G-CSF indirectly modulates DC function in humans, by inducing hepatocyte growth factor (HGF), IL-10, and interferon (IFN)-α, and mobilizes type 2 DCs (DC2) [44]. Overall, the available data imply that G-CSF-mobilized cell therapy products may be intrinsically less capable of inducing uncontrollable GVHD.

However, published evidence suggests that G-CSF might exert protective effects when given to haematopoietic stem cell (HSC) donors but not to HSCT recipients. In mice, the transplantation of G-CSF-mobilized splenocytes was associated with a significant reduction in tumour necrosis factor (TNF)-α and lipopolysaccharide (LPS) production and in GVHD score, translating into improved survival. In contrast, the administration of G-CSF to recipient animals exerted no effect on acute GVHD-related survival or in vivo TNF-α production in the absence of G-CSF pretreatment of the donor, indicating that G-CSF effects on the donor rather than on the recipient mice might account for the GVHD improvement [45].

Associations between immune cell subsets in G-CSF-mobilised grafts and clinical outcomes, including GVHD occurrence, were reported in patients receiving PBSC transplantation [46]. The frequencies of activated NK cells and Natural Killer T (NKT) cells were correlated with a significantly lower risk of acute GVHD. By contrast, late activated, HLA-DR-expressing T cells were associated with a significantly higher risk of acute and chronic GVHD. Intriguingly, the frequency of CD34-expressing monocytes in G-CSF-mobilised allografts inversely correlates with the incidence of acute GVHD, suggesting that in vitro-expanded G-CSF-mobilised monocytes might be used as GVHD therapeutics [47]. In the context of MUD HSCT, the intra-graft content of monocytic myeloid-derived suppressor cells (MDSCs), which are expanded as a result of G-CSF administration, was the only predictor of acute GVHD [48]. The cumulative incidence of acute GVHD at 180 days after transplantation for recipients receiving monocytic MDSC doses below and above the median was 63% and 22%, respectively. However, monocytic MDSCs had no apparent impact on relapse rates or TRM rates. There is also evidence that the administration of G-CSF-primed bone marrow grafts shares the advantages of G-CSF-mobilised peripheral blood grafts without being associated with increased risk of GVHD [49]. Direct comparisons of the immunological properties of G-CSF-mobilised blood and G-CSF-primed bone marrow samples indicate that G-CSF-primed bone marrows have reduced T-cell cytokine production, lower expression of CD28 costimulatory molecules on T cells, lower DC content, and diminished proliferation capacity [50].

Recently, a transplantation protocol (GIAC) involving sequential, in vivo modulation of T-cell functions in both recipients and donors was developed in China [51], the main elements of which stand for donor treatment with G-CSF (G), intensified immunologic suppression (I), anti-human thymocyte immunoglobulin for GVHD prevention (A), and the combination (C) of peripheral blood and bone marrow transplantation. This approach resulted in high rates of engraftment and similar incidence of GVHD as that observed in allogeneic HSCT from MRD, and was inspired by previous data from the same group showing that the combined use of G-CSF-primed BM and peripheral blood HSCs in proportions ranging from 2:1 to 1:2 maintained T-cell hyporesponsiveness and polarization towards a Th2 phenotype [52].

Taken together, these studies suggest that immune modulation by G-CSF might be used to affect graft composition and to prevent GVHD.

### 3.2. Low and Ultra-Low Dose IL-2

Treatment with IL-2 is a promising approach to expand Treg cells and NK cells in HSC donors. Ultra-low dose (ULD) IL-2 has been given to 21 healthy donors as an immune-modulating agent with the aim of preventing GVHD after HSCT [53]. Safety, dose level, and immune signatures were evaluated after the administration of 50,000 to 200,000 units/m^2^/day IL-2 for 5 consecutive days. The treatment was well tolerated and was associated with an increase of Treg cells with potent suppressive activity, as well as of CD56^bright^ NK cells with enhanced IFN-γ release. IFN-γ-induced protein 10 (IP10) was increased in the serum. Gene expression profiling revealed a significant change in a restricted set of genes, including *FOXP3* and *IL-2RA*.

Low-dose IL-2 has been tested in a controlled, open-label randomized trial that included 90 recipients of allogeneic HSCT [54]. Patients in the IL-2 arm received a subcutaneous injection of low-dose IL-2 (1 × 10^6^ U/d) on day 60 after HSCT for 14 consecutive days, followed by a 14-day hiatus. Detection of the minimal residual disease occurred more frequently in IL-2-treated patients compared with the control arm (36% vs. 15%). The cumulative incidence of moderate-to-severe chronic GVHD was significantly lower in IL-2-treated patients compared with controls (33% vs. 57%), leading to significantly higher 3-year GVHD-free PFS rates (47% in IL-2 treated patients vs. 31% in controls). In line with previous findings in patients treated with IL-2 for steroid-refractory chronic GVHD [55], blood Treg cells, NK cells, and NK-cell cytotoxicity were increased in IL-2-treated subjects between 3 and 6 months after HSCT [54].

## 4. T-Cell Depleted (TCD) Haploidentical Haematopoietic Stem Cell Transplantation (HSCT) as a Platform for Adoptive Immunotherapy

Many groups are currently evaluating adoptive immunotherapy with transplant donor-derived T cells to treat and/or prevent leukaemia relapses and drug-resistant infectious complications [11,56]. Investigators at Memorial Sloan Kettering Cancer Centre, New York, have pioneered the use of in vitro expanded T cells specific for peptide epitopes of the Wilms Tumour 1 (WT-1) protein in patients with WT-1^+^ hematologic malignancies [11]. WT-1 is a suitable target for adoptive immunotherapy being differentially expressed in over 70% of AMLs and myelomas and being also expressed at high levels in advanced MDS. Importantly, in transplant recipients treated with unselected donor lymphocytes for the relapse of AML or myeloma, expansion of WT-1 specific T-cells closely correlates with the eradication of tumour cells and the achievement of complete remission [57]. WT-1 specific T cells generated from normal HSCT donors were administered at escalating doses (3.8 × 10^8^ to 3.3 × 10^9^/m^2^) to 11 patients with WT-1^+^ leukaemia, MDS, or myeloma in disease relapse post-transplantation [58]. At high doses, WT-1 specific T-cells expanded in vivo, preferentially localised to the bone marrow compartment and were detected in the blood for more than 7–14 months, with the induction of complete remissions.

### 4.1. Suicide Gene-Transduced T Cells to Provide Graft-Versus-Leukaemia (GVL) Responses without GVHD

Several groups are using suicide gene-transduced donor T cells to control GVHD, while promoting post-transplantation immune reconstitution. A recent phase I/II multi-centre clinical trial of transplantation with TCD haploidentical HSCs explored the potential benefit of infusing donor lymphocytes genetically engineered to express the human herpes simplex virus thymidine kinase type 1 (HSVtk) suicide gene, with the aim of boosting immune reconstitution and improving disease control in patients with high-risk leukaemia [59]. Gene-modified cells were infused starting at day 28 after HSCT, with an initial dose of 1 × 10^6^ cells/kg. In the absence of GVHD and immune recovery, a second dose of 1 × 10^7^ cells/kg, a third dose of 1 × 10^6^ cells/kg plus subcutaneous recombinant human IL-2 (rhIL-2), and a fourth dose of 1 × 10^7^ cells/kg plus rhIL-2 were administered at monthly intervals. Collectively, no acute or chronic events related to the gene-transfer procedure were observed. Engraftment was documented in 22 out of the 28 patients infused, and CD3^+^ T-cell counts >100/µL were achieved by day 75 after HSCT [59]. Importantly, CMV- and EBV-specific, IFN-γ-producing T cells were detected at the time of immune recovery. Anti-viral responses progressively normalized in patients who attained immune recovery. The infusion of HSVtk cells was correlated with the development of GVHD in 10 out of 22 immune-reconstituted patients. Treatment with ganciclovir translated into a significant reduction of circulating HSVtk cells, but not CD3^+^ T cells, reassuring about the lack of impact of ganciclovir on long-term immune recovery. Collectively, the overall survival in patients with de novo acute leukaemia transplanted in any complete remission was 49% at 3 years [59].

A different gene construct was used for the transduction of donor T cells by Di Stasi and co-workers [17]. Human caspase-9 (C9) was fused to a modified human FK506-binding protein whose dimerization, in the presence of a synthetic bioinert drug, was shown to trigger the activation of C9 and death of the cells expressing the construct [17]. ∆CD19 was used as a “selectable marker” to isolate transduced T cells that were then administered to 5 patients after haploidentical HSCT with purified CD34^+^ cells. Modified T cells displayed a CD3^+^∆CD19^+^ phenotype and were detectable in vivo within 3–7 days after the first infusion. Concomitantly with the expansion of gene-modified T cells, mild GVHD of the skin occurred in 4 of 10 patients [60]. Each patient with GVHD received one infusion of the dimerizing drug AP1903, which induced a more than 90% decline of blood transgenic cells within 30 min [17]. GVHD-associated abnormalities were resolved within 24 h after infusion and without additional immune suppressive therapy. Gene-modified T cells were detected in stable numbers for more than 1 year and mainly comprised polyclonal CD3^+^CD19^+^ T cells that also contained virus-reactive T cells. In line with this finding, no patient experienced reactivation of CMV, EBV, HAdV, BK virus, TCD Haploidentical HSCT As a Platform for Adoptive Immunotherapy, and *Aspergillus* after T-cell infusion and treatment with AP1903. The authors confirmed these findings in patients treated with iC9-DLI without any prior allodepletion step [61]. Safety switch-modified T cells persisted in vivo for more than 2 years and accelerated the recovery of endogenous T cells, including CD4^+^ T cells of thymic origin [60].

Although further studies are required to confirm efficacy, the infusion of suicide-gene transduced T cells is a very promising approach to enhance immune recovery without inducing GVHD [61].

### 4.2. Virus-Specific T Cells

Viral infections continue to account for substantial post-transplant morbidity and mortality after allogeneic HSCT, as reviewed elsewhere [62]. Immunotherapeutic strategies are increasingly being exploited to prevent and treat viral infections when anti-viral drugs are ineffective or cause excessive toxicity. Anecdotal reports suggest the safety and tolerability of infusing virus-specific T cells activated ex vivo using pools of overlapping peptides [56]. Virus-specific T cells stimulated with overlapping peptides derived from the immunodominant HAdV5 hexon protein (MACS GMP Peptivator^TM^ AdV5 hexon, Miltenyi Biotec) were recently used to treat disseminated HAdV infection after TCD haploidentical HSCT. The IFN-γ-secreting T cells are then labelled and magnetically enriched using the Cytokine Secretion System and the Clini-MACS^TM^ device. The adoptive transfer of HAdV-specific T cells was safe and not associated with any adverse event, including alloreactivity against the recipient’s tissues. Recovering CD4^+^ and CD8^+^ T cells mostly displayed a CD45RO^+^ memory phenotype, released IFN-γ in response to HAdV-derived peptides, but lacked in vitro responses against other dominant HAdV antigens not used for T-cell activation, as well as responses to other viral pathogens, such as CMV and EBV [56].

The Memorial Sloan Kettering Cancer Research Centre and Baylor College of Medicine Groups have established consortia to foster the implementation of multi-centre clinical trials of banked third party T cells for EBV, CMV, and other life-threatening viral infections complicating HSCT [63].

## 5. Novel Strategies for Controlling GVHD

While TCD strategies remain a cornerstone for GVHD control, other avenues are being explored to reduce GVHD while preserving anti-viral and anti-tumour responses. Targeting epigenetic modifiers such as acetyl and methyl-transferases, micro-RNAs, inhibiting Notch signalling and mitochondrial ATP-ase, inhibiting protein kinase-C, and JAK/STAT signalling have all emerged as potential therapeutic targets for GVHD control. Earlier studies using histone deacetylase inhibitors (HDACs) such as suberoylanilide hydroxamic acid (SAHA/vorinostat) showed that these drugs can mitigate the effect of GVHD by impairing the function of host APCs [64]. Treatment of DCs with HDACs also led to the induction of the tryptophan catabolising enzyme indoleamine 2,3-dioxygenase-1 (IDO1), which is an inhibitor of DC and T-cell function [65]. Other studies in mouse models have shown that the inhibition of HDAC6 (a non-histone deacetylase) specifically abrogates CD8 T-cell function and significantly reduces GVHD-like manifestations [66]. Similarly, the inhibition of histone methylation using DZNep (3-deazaneplanocin A) resulted in the apoptosis of activated alloreactive T cells and diminished tissue damage and injury in the host [67,68].

Notch signalling is one of the key regulators of T-cell and DC development. Targeting Notch signalling to control murine GVHD has attracted considerable interest. The inhibition of Notch in T cells resulted in reduced pro-inflammatory cytokine production without compromising immune cell proliferation [69]. Gatza et al. also showed that targeting mitochondrial ATPase could reduce the frequency of alloreactive T cells in GVHD without affecting T-cell responses [70]. PKC-α is a key regulator of T-cell signalling through its interaction with several transcription factors, including NF-AT. Small-molecule inhibition of PKC-θ and PKC-α has been successfully pursued in murine models of GVHD [71]. Several other potential candidates, such as JAK-STAT inhibitors [72], miRNA inhibitors [73] and aurora kinase-A inhibitors [74] are currently being evaluated. However, the clinical efficacy of the above candidate drugs has yet to be substantiated in clinical trials.

## 6. Conclusions

Haploidentical HSCT could be offered to patients with an indication for allogeneic HSCT, but we do not have a MRD or a MUD available within a reasonable time frame. TCD haploidentical HSCs can secure consistent engraftment without an increase in relapse in patients transplanted for acute leukaemia. Unquestionably, T-cell depletion is an efficient tool for preventing acute and chronic GVHD after haploidentical HSCT. However, immunological reconstitution may be delayed, requiring cellular therapy approaches to boost the recovery of anti-leukaemia and pathogen-specific immunity. The continuous availability of haploidentical donors will allow us to address the issues of delayed immune reconstitution and post-transplantation leukaemia recurrence by adoptively transferring non-alloreactive T cells, either genetically modified (iC9 or HSVtk) or specifically selected. Innovative protocols for graft engineering, including the immunomagnetic depletion of GVHD-inducing CD45RA-expressing T cells, are currently being tested with varying degrees of success.

In vitro T-cell depletion protocols are laborious and expensive, compared with other approaches that are being successfully implemented in the clinic, including the administration of PTCy. Randomised clinical trials to assess the superiority of each approach have not yet been conducted.

In conclusion, the generation of “designer grafts” will hopefully pave the way to safer HSCT approaches in patients with malignancies or will serve as a platform for tolerance induction in patients’ with autoimmune diseases.

## Figures and Tables

**Table 1:** Clinical trials with TCR-αβ/CD19-depleted haematopoietic stem cells (HSCs).

Patients	Disease	Graft-versus-Host Disease (GVHD) Prophylaxis	Acute/Chronic GVHD	TRM	EFS(DFS)/OS	Reference
28	HR-AML	FK506, MTX	39%/30%	10%	60%/67% (2 years)	[19]
37	PID	FK506, MTX; FK506, MMF; CYA, MTX	22%	3.3% (27% GF)	96.7% (15 months)	[20]
41	AL	MMF	10%/9%	N.A.	21/41 patients alive after 1.6 years	[21]
23	Non-malignant	None	13%/0%	9.3%	91% (2 years)	[16,22]
34	HR-AL	N.A.	5.9%/6.1%	14.7%	42%/54% (1 year)	[23]
80	AL	None	30%; no extensive chronic GVHD	5%	71%/72% (5 years)	[24]

Legend: HR-AML = high-risk acute myeloid leukaemia; CYA = cyclosporine-A; MTX = methotrexate; DFS = disease-free survival; EFS = event-free survival; OS = overall survival; GF = graft failure; PID = primary immune deficiencies; AL = acute leukaemia; HR-AL = high-risk acute leukaemia; MMF = mycophenolate mofetil; N.A. = not available; TRM = transplantation-related mortality.

**Table 2:** Clinical trials with CD45RA T-cell depletion.

Patients	Disease	Graft-Versus-Host Disease (GVHD) Prophylaxis	Acute/Chronic GVHD	TRM	EFS(DFS)/OS	Reference
35	High-risk leukaemia	Tacrolimus	66%; 9%	9%	70%/78% (2 years)	[29]
8	Solid tumours	Sirolimus	No acute GVHD or GF	1 patient died of sinusoidal obstruction syndrome	N.A. (median follow-up was 184 days)	[34]
17	Haematological malignancies	Sirolimus and MMF	17.6% grades III–IV acute GVHD/6 patients with signs of oral or skin chronic GVHD	11.7%	76.5% of patients alive at a median of 223 days after haematopoietic stem cell transplantation (HSCT)	[35]

DFS = disease-free survival; EFS = event-free survival; OS = overall survival; TRM = transplantation-related mortality; GF = graft failure; MMF = mycophenolate mofetil; N.A. = not available.
